# Total Hip Replacement in Sickle Cell Disease Patients with Avascular Necrosis of Head of Femur: A Retrospective Observational Study

**DOI:** 10.1007/s43465-021-00394-6

**Published:** 2021-03-28

**Authors:** Mohammed Lafi Al-Otaibi, Shah Waliullah, Vineet Kumar

**Affiliations:** 1 Department of (Orthopedics) Surgery, College of Medicine, King Khalid University, Abha, Kingdom of Saudi Arabia; 2 Department of Orthopaedic Surgery, King Georges’ Medical University, Lucknow, India; 3 Ram Manohar Lohia Institute of Medical Sciences, Lucknow, India

**Keywords:** Sickle cell disease, Hip joint congruency, Total hip replacement, Harris hip score

## Abstract

**Background:**

Femoral head avascular necrosis leads to osteoarthritis of the hip joint and affects its functional capacity in sickle cell disease patients. The functional outcomes of total hip replacement (THR) on patients with congruous joints who underwent hip replacement after having a failed joint preservation surgery are unknown. This study aimed to compare the functional outcomes of THR in patients with sickle cell disease having avascular necrosis with and without loss of hip joint congruency.

**Methods:**

This retrospective study included 35 patients (age, 20–52 years; 18 males and 17 females) who underwent uncemented THR. Patients were divided into Group-A (*n* = 18, good hip joint congruency) and Group-B (*n* = 17, obliterated hip joint congruency). The Harris Hip Score (HHS) was used to assess functional outcomes. All patients were followed up at 6-weekly intervals then 6-monthly intervals.

**Results:**

The mean follow-up period was 8.26 ± 3.01 years. The mean preoperative HHSs of Group-A and Group-B were 45.22 ± 3.021 and 25.94 ± 4.437, respectively. Postoperatively, a subsequent increase in HHS was found in both groups, and a significant difference between the groups was observed at 6 weeks (*p* < 0.0001*) and 1 year (*p* < 0.0006*). Interestingly, HHS was not significantly different (*p* = 0.0688) at 5-year follow-up between the groups. The differences in HHS within the group at each subsequent follow-up were also statistically significant (ANOVA, *p* < 0.0001*).

**Conclusion:**

A significant improvement was observed with THR in both groups. Nevertheless, the flattened hip joint congruency group showed significantly better HHS improvements than the normal congruency groups. These findings may aid in the decision-making capabilities of the surgeons.

## Introduction

Sickle cell disease is an autosomal recessive genetic disorder characterized by abnormal sickle hemoglobin production with decreased red blood cells’ pliability. It results in clogging of blood vessels, leading to ischemia and infarction of the affected tissue [1]. Though it is endemic throughout tropical Africa and the Middle East region, populations’ mobility has turned it into a global disease [2]. Bone involvement is one of the common clinical manifestations of sickle cell disease and ranges from acute painful vaso-occlusive crisis to chronic disability such as avascular necrosis. Sickle cell disease involves various orthopedic complications including osteonecrosis, pathological fractures, osteomyelitis, and septic arthritis [3].

Femoral head is the most typical site for avascular necrosis in sickle cell disease, followed by shoulder, knee, and other small joints [4]. Avascular necrosis of the femoral head leads to osteoarthritis of the hip joint and affects its functional capacity. Treatment of osteonecrosis requires early diagnosis and timely intervention to prevent morbidity and mortality related to late diagnosis. To improve functional capacity, the majority of orthopedic surgeons recommend total hip replacement [5, 6].

There are many skeletal issues related to the hip joint in sickle cell disease patients, including abnormal metaphyseal femoral morphology with thin cortices and trabeculae, low bone density, and medullary hyperplasia. Irregular bone sclerotic areas can also obliterate the femoral canal and cause loss of hip joint congruency [7]. Sometimes, thinning of the femoral cortical lining inside the outer cortex appears as a femur within a femur [8].

In our clinical practice, we observed that femoral head congruency strongly correlated with functional improvement after total hip replacement. In patients with loss of joint congruency, the clinical picture was characterized by greater suffering in terms of pain and limitation of motion due to arthritis. Total hip replacement decreases these clinical features dramatically and improves the overall patient conditions. However, there is a subset of patients within the early stages of avascular necrosis with congruous joint presenting with pain and not responding to joint preservation procedures like core decompression. Total hip replacement can alleviate symptoms and improve the quality of life in such patients. Nevertheless, to date, there has been a dearth of literature pertaining to this subset of patients with congruous joints who failed joint preservation surgery initially and had undergone a hip replacement. Thus, in the current study, we retrospectively analyzed functional outcomes in patients with sickle cell disease having avascular necrosis with and without loss of hip joint congruency.

## Materials and Methods

### Study Design

The study was approved by our Institutional Review Board (HAPO-06-B001). We conducted a retrospective case series with all patients recruited from a tertiary care central hospital (Aseer Central Hospital).

**Fig. 1:**
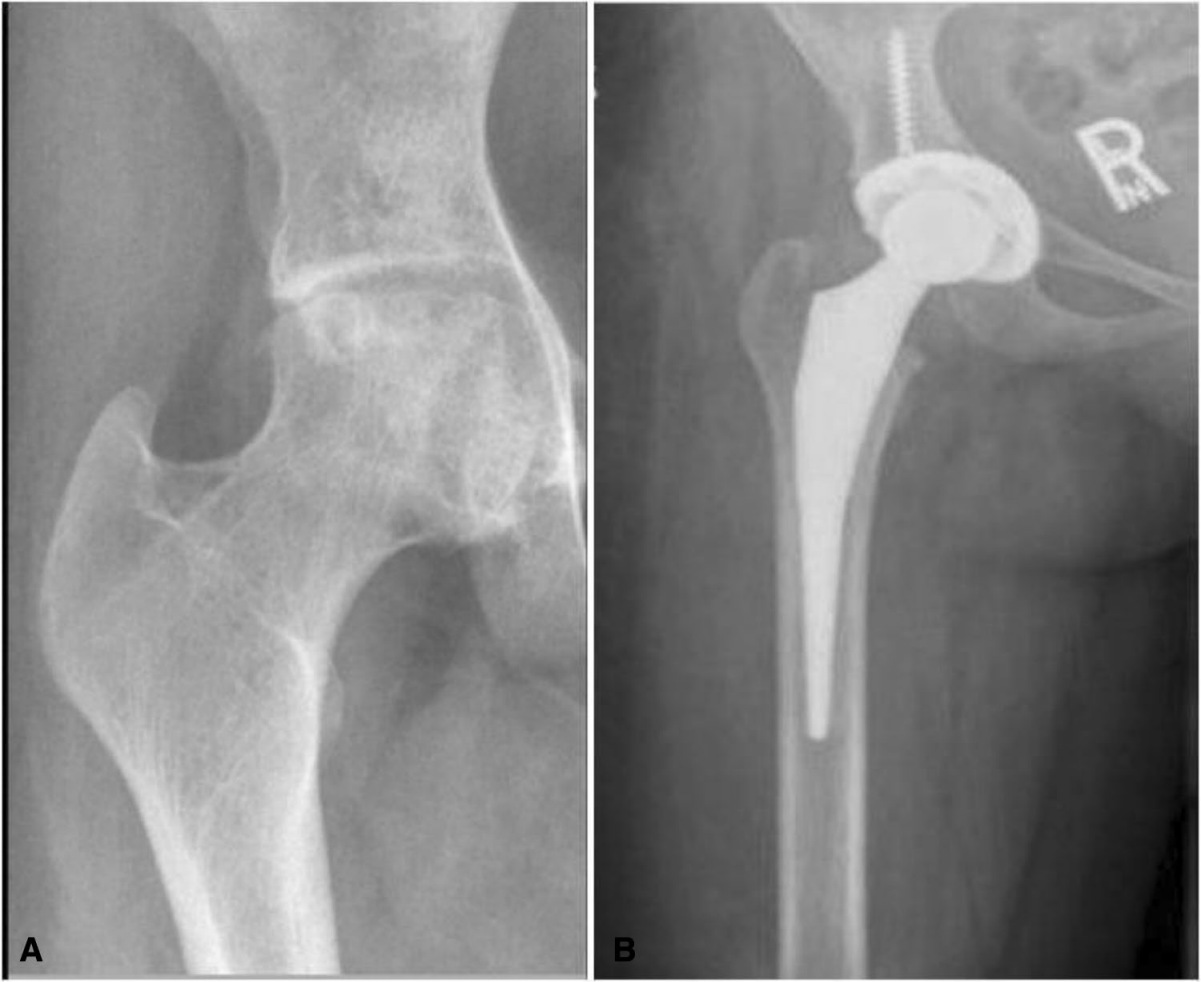
**a** Hip joint with subchondral collapse with a triangular shape and loss of acetabular congruency pre-operatively. **b** Post-operative hip joint with prosthesis

**Fig. 2:**
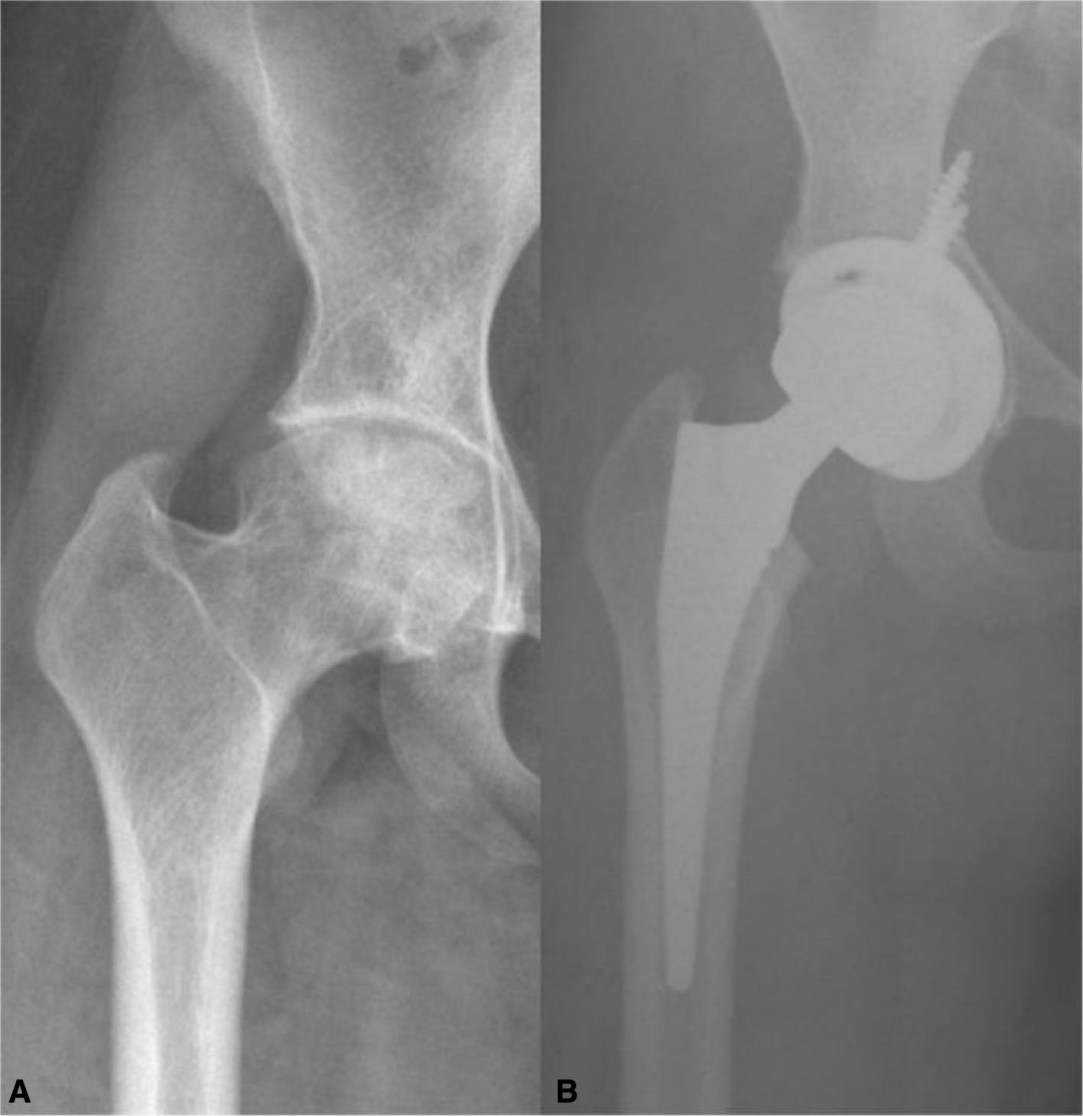
**a** Hip joint with preserved acetabular congruency pre-operatively. **b** Post-operative hip joint with prosthesis

**Fig. 3:**
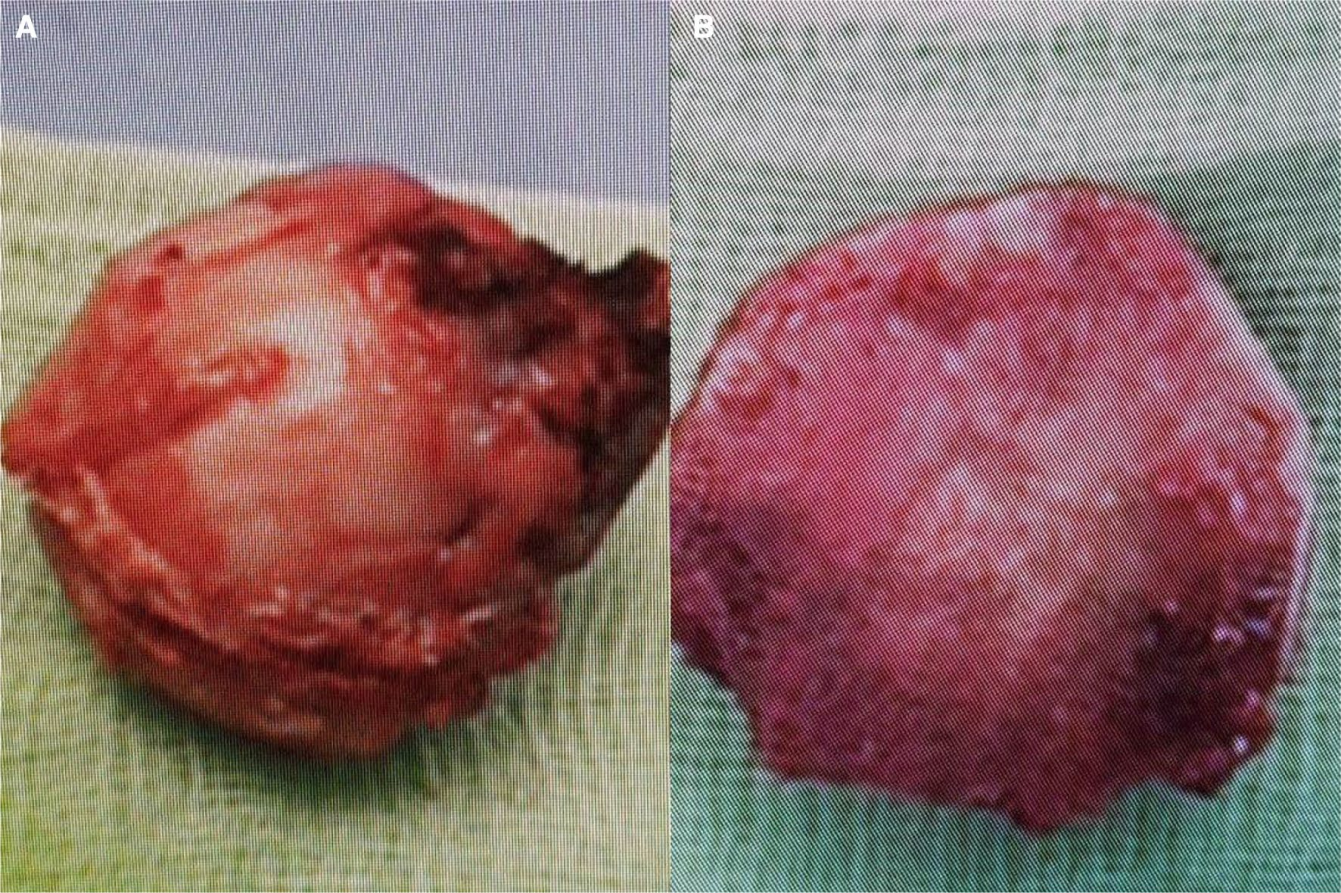
Post-surgical observation of the head of the femurs. **a** Roundhead of the femur. **b** Triangular head of the femur

A total of 35 patients, aged 20–52 years (18 males and 17 females) underwent total hip replacement secondary to avascular necrosis of femoral head due to sickle cell disease, between 2008 and 2015 as per the institutional records retrieved from the database. All the patients had typical clinical manifestations of sickle cell disease with multiple admissions for vaso-occlusive sickle cell crises. The detailed records of their clinical, radiographical, and hematological data were obtained. All patients had undergone preoperative X-rays and magnetic resonance imaging to quantify infarct areas and were classified as per Ficat-Arlet grading. Patients were divided into two groups. The first group (Group-A) had 18 patients with good hip joint congruency (Ficat- Arlet grade III) and the second group (Group-B) had 17 patients with obliterated hip joint congruency (Ficat-Arlet grade IV). In Group-A, although the patients had congruous hip joint radiologically, magnetic resonance imaging showed an infarct area greater than 30%. They all underwent prior joint preservation procedures (core decompression) but failed to respond to the treatment in terms of pain relief. In Group-B, all patients had incongruous hip joint with arthritic changes. Uncemented total hip replacement was performed in all cases (Figs. 1, 2 and 3). We excluded patients who had bilateral involvement with single-side loss of acetabular congruency. Patients' information was kept confidential.

### Peri-Operative Care

All patients were admitted 3 days before surgery and were evaluated by a hematologist before undergoing an exchange transfusion with the goal of reaching a preoperative hemoglobin concentration of at least 10 g/dl and Hb S concentration < 30%. All patients were operated under either spinal or general anesthesia. Adequate hydration and oxygenation were maintained in the patients to avoid sickle-triggering factors such as hypothermia and dehydration before and during the procedure. Preoperative antibiotics were given before induction and continued for 3 days postoperatively as per the institutional policy for sickle cell patients.

### Surgical Technique

All procedures were done through the lateral approach. The incision was made 5 cm proximal to the tip of the greater trochanter longitudinal incision centered over the greater trochanter’s tip and extended about 8 cm down the line of the femur. The superficial dissection split the fascia lata and retracted it anteriorly to expose the tendon of the gluteus medius. Using sharp dissection, we detached the fibers of the gluteus medius that were attached to fascia lata. Deep dissection split the fibers of the gluteus medius longitudinally starting at the middle of the greater trochanter that did not extend more than 3–5 cm above the greater trochanter to prevent injury to the superior gluteal nerve, extending the incision inferior through the fibers of the vastus lateralis. This developed an anterior flap in the anterior gluteus medius from the anterior greater trochanter with its underlying gluteus minimus. The anterior part of the vastus lateralis required sharp dissection of the muscles off the bone or lifting of small flecks of the bone when possible, to expose the anterior joint capsule. The following dissection anteriorly along the greater trochanter and on to the femoral neck led to the capsule of the gluteus minimus that was released from the anterior greater trochanter, allowing easy dislocation of the hip joint. Adequate exposure and meticulous soft-tissue handling were done to avoid complications or fracture. Extra precautions were taken while femoral stem preparation in patients with sclerotic and narrowed femoral canal. Sequential reaming over guidewire was done to prevent femoral stem perforation.

During the postoperative period, the patients in both groups underwent standard physical therapy protocol for total hip replacement including patient education, pain management, range of motion, and muscle strengthening exercises. Patients were allowed partial weight-bearing for the initial 6 weeks, followed by full weight-bearing. The patients were discharged from the hospital 10–21 days post-surgery. Harris Hip Score (HHS) [9, 10] was used to assess the functional outcomes. All patients were followed up at 6-week intervals and then 6-monthly intervals to determine functional outcomes clinically and radiographically. Radiographs were assessed for any evidence of loosening, dislocation, or heterotopic ossification. Failure was defined as the need for revision replacement surgery due to surgical complications, infection, loosening, or dislocation. Patients were seen by a hematologist and the study author at every follow-up, and medical and surgical complications were documented.

### Statistical Analysis

SPSS version 22.0 (IBM, Armonk, NY, USA) was used to analyze the data. Descriptive statistics were used to calculate the arithmetic means and standard deviations for the demographic variables and outcome measures. Chi-squared and student’s *t* tests were used as the tests of significance at a 5% level of significance.

## Results

A total of 35 patients (mean age, 34.914 ± 8.38 years) underwent a unilateral total hip replacement. All patients were followed up for a mean period of 8.26 ± 3.01 years (range 5–12 years). Three patients died due to causes unrelated to the surgery. The mean age of Group-A and Group-B patients were 32.22 ± 6.217 and 37.76 ± 9.562 years, respectively. The majority of the patients underwent a right-sided total hip replacement, and there were no significant differences observed in demographical comparisons between Group-A and Group-B (Table 1).

**Table 1:** Demographic characteristics

	Total [*n* = 35] Mean ± SD Median	Group-A [*n* = 18] Mean ± SD Median	Group-B [*n* = 17] Mean ± SD Median	*p* value
Age	34.914 ± 8.381 34	32.22 ± 6.217 32	37.76 ± 9.562 38	*t* = 2.02 *p* = 0.0533
Gender				
Male	18 (51.42%)	9 (25.71%)	9 (25.71%)	*χ* = 0.0084 *p* = 0.926
Female	17 (48.57%)	9 (25.71%)	8 (22.85%)	
Side				
Right	21 (60%)	11 (31.43%)	10 (28.57%)	*χ* = 0.0190 *p* = 0.890
Left	14 (40%)	7 (20%)	7 (20%)	

*SD* standard deviation

**Fig. 4:**
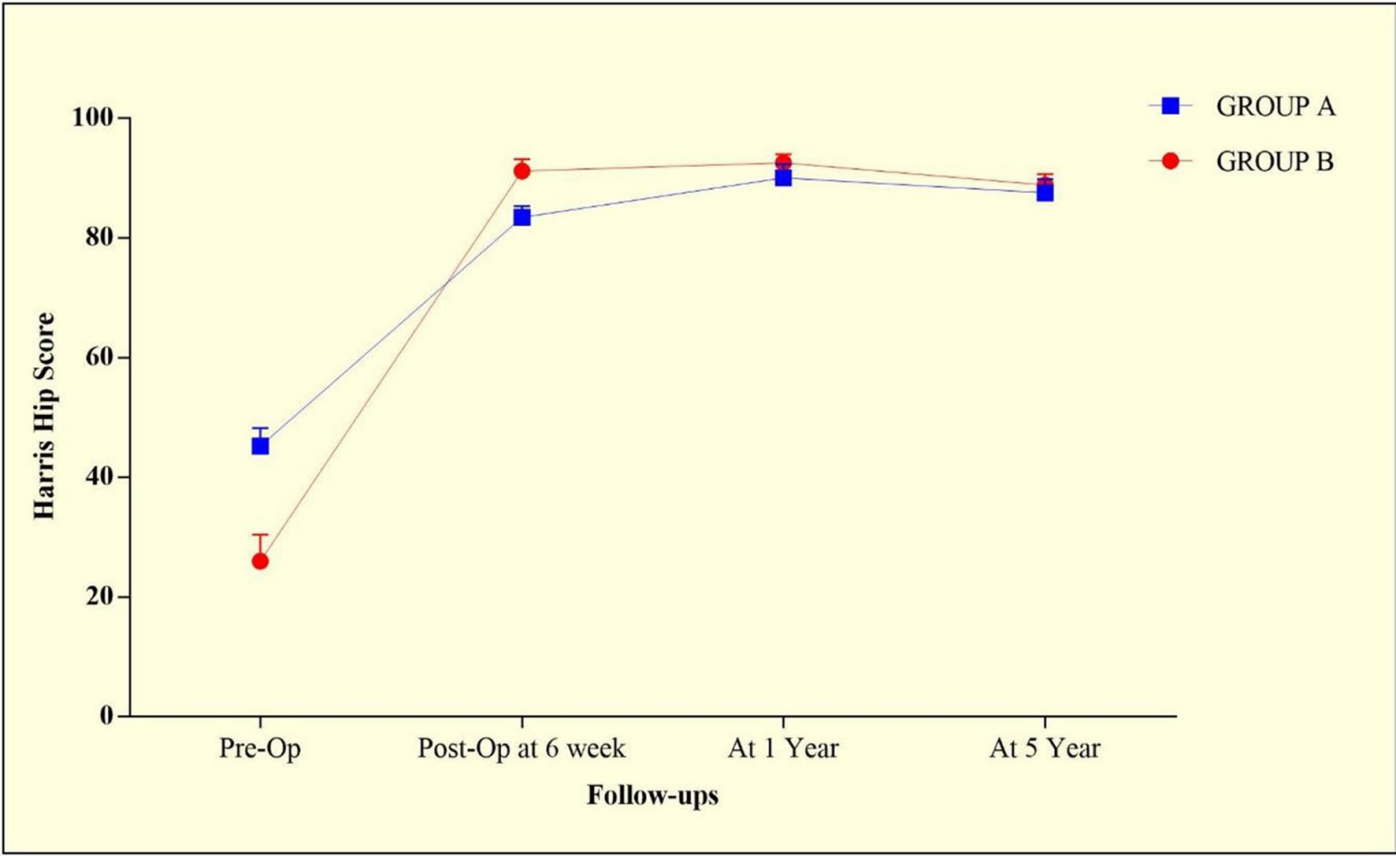
Graphical representation of Harris Hip Score evaluation at different follow-up periods. *Pre-OP* preoperatively, *Post-OP* postoperatively

The mean preoperative HHSs of Group-A and Group-B were 45.22 ± 3.021 and 25.94 ± 4.437, respectively, and the difference was statistically significant (*p* < 0.0001*). At follow-up, a subsequent increase in the HHS score was found in both groups, and the difference between the groups were statistically significant at 6 weeks (*p* < 0.0001*) and 1 year (*p* < 0.0006*) postoperatively. Interestingly, no significant difference (*p* = 0.0688) in the HHS was observed at 5 years of follow-up in both groups. Furthermore, the differences in the HHS within the group at each subsequent follow-up were also found to be statistically significant (ANOVA, *p* < 0.0001*) (Table 2, Fig. 4).

**Table 2:** Harris Hip Score evaluation at different follow-up

Harris Hip Score	Group-A [*n* = 18] Mean ± SD Median	Group-B [*n* = 17] Mean ± SD Median	*p* value
Pre-OP	45.22 ± 3.021 45	25.94 ± 4.437 24	*U* = 0 *p* < 0.0001*
Post-OP at 6 week	83.44 ± 1.917 83	91.18 ± 2.007 92	*U* = 0 *p* < 0.0001*
At 1 year	90 ± 2.301 89.5	92.53 ± 1.419 93	*U* = 54 *p* < 0.0006*
At 5 year	87.5 ± 2.285 87.5	88.82 ± 1.845 89	*U* = 99 *p* = 0.0688
ANOVA test	*F* = 1370 *p* < 0.0001*	2464 *p* < 0.0001*	

*p < 0.05

*ANOVA* Analysis of variance, *Pre-OP* preoperatively, *Post-OP* postoperatively; *SD* standard deviation

Overall survival in the present study was 94.28%, and there was no significant difference in survival in both groups at 5-year follow-up. Nineteen patients (ten in Group-A and nine in Group-B) were admitted for sickle cell crisis during their follow-up and were managed effectively. Intra-operatively, two patients in Group-A had a femoral fracture during stem insertion, managed by cerclage wiring. One patient in Group-B developed femoral stem perforation while reaming which was managed by a long femoral stem. Five patients (three in Group-A and two in Group-B) developed superficial infections and were managed by extended antibiotics duration. In Group-A, one patient developed a deep infection at 2 years follow-up and required a two-stage revision total hip replacement. One patient in Group-B had aseptic loosening of the stem in the 7th year and underwent revision. Two patients in Group-A and one patient in Group-B developed Brooker grade II heterotopic ossification [11].

## Discussion

To our knowledge, this is the first study to compare the effect of femoral head congruency on the functional outcomes of total hip replacement in patients with sickle cell disease. A unique strategy of comparing the effect of loss of congruency on functional outcomes of total hip replacements in patients with sickle cell disease was employed. Both groups had significant improvements in functions after total hip replacement which is similar to the data reported in many previous studies [12–14]. Interestingly, we found that the group with incongruous head i.e., with the collapse of the femoral head (Ficat-Arlet grade IV) showed better improvements in functional performance during the first year of the follow-up than the other group with preserved head contour (Ficat-Arlet grade III) as demonstrated by HHS. However, in long-term follow-up (at 5 years), the results were comparable between the groups. The reason for this finding may be that the patients with incongruous joint already had hip arthritis leading to disability and pain as evident by lower preoperative HHS in comparison to those in patients with a congruous hip. On subjecting these patients with incongruous hip or advanced stage avascular necrosis to total hip replacement, a significant improvement in terms of HHS was observed.

We observed that 54.28% of the patients required readmission due to the medical complications, irrespective of congruency status, however, we did not observe any significant difference in terms of surgical complications between the groups. Overall, the survival rate at the most recent follow-up (5 years postoperatively) was 94.29%. The study findings are comparable to the studies by Azam et al. [14], who showed a 92.6% survival rate at a mean follow-up period of 7.5 years among 67 sickle cell disease patients who underwent uncemented total hip replacements and Ilyas et al. [6], who observed a survival rate of 94.1% at 15-year follow-up after uncemented hip arthroplasty for avascular necrosis in 101 sickle cell disease patients.

We observed a superficial infection rate of 14.2% and a deep infection rate of 2.8% in this case series. Superficial infection was managed effectively by an extended period of antibiotics as per the culture and sensitivity, whereas deep infection required two-step hip replacement. Sickle cell patients are prone to infections because of low immunity, so it is imperative to take adequate measures to prevent perioperative infections in these patients. The deep infection rate reported in our study is less as compared to 3.76% as reported by Ilyas et al. [6] and 5.7% as reported by Farook et al. [12].

Although we did not observe any dislocations, aseptic femoral stem loosening was seen in 2.8% of our cases and all of them were finally managed by revision arthroplasty. The complication of aseptic loosening is not so uncommon in cases of avascular necrosis due to sickle cell disease as Issa et al. [15] also reported a 5% revision rate due to the said complication.

Of all our cases who had undergone an uncemented total hip arthroplasty, Brooker grade II heterotopic ossification was found in 8.5% of the subjects. This result is in accordance with Illyas et al. [6] who reported a 9% incidence of heterotopic ossification in their study on sickle cell patients with an uncemented total hip replacement, while Hernigou et al. [5] reported a 4% rate with cemented total hip replacement.

The hip joint lacks sphericity and congruency at birth. It also lacks stability and is susceptible to subluxation and dislocations. As a result of loading and musculoskeletal changes, a deeper spherical acetabular cavity forms that improves joint stability [16]. Many developmental conditions characterized by a lack of hip joint congruency may lead to osteoarthritis of the hip. This is the primary explanation, and the secondary reasons for osteoarthritis could be femoroacetabular impingements [17]. It was speculated that patients with loss of congruency would have more clinical abnormalities and that total hip replacement in these patients would result in significant improvements in function. The results of the present study support this hypothesis.

Our study has particular limitations. Due to the smaller sample size and single-centric analysis, results can not be generalized. We have not compared bone grafting procedures, proximal femoral osteotomies recommended for Ficat Arlet stage III with total hip replacement. In the future multicentric, randomized comparative studies can provide robust evidence.

## Conclusion

Improvements in the flattened hip joint congruency group were better than those in the normal hip joint congruency group. We conclude that preoperative hip joint congruency significantly affects the functional improvements in total hip replacements in sickle cell disease patients. Our findings may help in better patient selection for total hip arthroplasty in sickle cell anemia associated avascular necrosis of the femoral head.
